# Morphogenesis-regulated localization of protein kinase A to genomic sites in *Candida albicans*

**DOI:** 10.1186/1471-2164-14-842

**Published:** 2013-12-01

**Authors:** Alida Schaekel, Prashant R Desai, Joachim F Ernst

**Affiliations:** Department Biologie, Molekulare Mykologie, Heinrich-Heine-Universität, Düsseldorf, Germany; Manchot Graduate School Molecules of Infection, Heinrich-Heine-Universität, Düsseldorf, Germany

**Keywords:** *Candida albicans*, Protein kinase A, Tpk1, Tpk2, Efg1, Morphogenesis

## Abstract

**Background:**

The human fungal pathogen *Candida albicans* is able to undergo morphogenesis from a yeast to a hyphal growth form. Protein kinase A (PKA) isoforms Tpk1 and Tpk2 promote hyphal growth in a signalling pathway via the transcription factor Efg1.

**Results:**

*C. albicans* strains producing epitope-tagged Tpk1 or Tpk2 were used in genome-wide chromatin immunoprecipitation on chip (ChIP chip) to reveal genomic binding sites. During yeast growth, both PKA isoforms were situated primarily within ORFs but moved to promoter regions shortly after hyphal induction. Binding sequences for Tpk2 greatly exceeded Tpk1 sites and did not coincide with binding of the PKA regulatory subunit Bcy1. Consensus binding sequences for Tpk2 within ORFs included ACCAC and CAGCA motifs that appeared to bias codon usage within the binding regions. Promoter residency of Tpk2 correlated with the transcript level of the corresponding gene during hyphal morphogenesis and occurred near Efg1 binding sites, mainly on genes encoding regulators of morphogenesis*.*

**Conclusions:**

PKA isoforms change their genomic binding sites from ORF to promoter regions during yeast-hyphal morphogenesis. Tpk2 binds preferentially to promoters of genes encoding regulators of cellular morphogenesis.

**Electronic supplementary material:**

The online version of this article (doi:10.1186/1471-2164-14-842) contains supplementary material, which is available to authorized users.

## Background

The fungus *Candida albicans* is an important cause of human disease, causing tenacious superficial and life-threatening systemic infections. Its virulence depends to a large extent on its ability to switch between a yeast and a hyphal growth form [1]. Environmental conditions favouring hyphal development include molecules of the human host acting as inducers, as well as physical parameters such as body temperature. Protein kinase A (PKA) isoforms Tpk1 and Tpk2 have crucial roles as signalling kinases because they mediate several adaptation responses to host contact [2–4]. In inducing conditions, cAMP is generated by adenylate cyclase (Cyr1) and triggers PKA activity by binding and removal of the inhibitory subunit Bcy1, which associates with Tpk1 and Tpk2 [5–7]. The cAMP-PKA pathway subsequently activates the Efg1 transcription factor, which represents the central hub controlling downstream events including morphogenesis and metabolic adaptation [1, 8–10]. Efg1 fulfills its morphogenetic functions by association with co-regulators Czf1, Flo8, Slf1 and Slf2 [11, 12]. Interestingly, in spite of their association with the same regulator protein Bcy1, both PKA isoforms exert specific environment-dependent functions with regard to hyphal morphogenesis [3] and Tpk2 but not the Tpk1 isoform mediates downregulation of *EFG1* expression early in hyphal induction [13].

PKA localization differs among species: in budding yeast the PKA holoenzyme is localized in the nucleus [14], whereas in fission yeast it resides in the cytoplasm [15] and in mammalian cells PKA catalytic subunits bind to anchoring proteins in different intracellular localizations [16, 17]. In spite of these differences, it appears that in all species important AGC kinase activities are needed in the nucleus. Increased cAMP levels lead to partial entry of PKA catalytic subunits into nuclei of fission yeast [15] and mammalian cells [16, 17]. In *C. albicans*, phosphorylation of the Tpk2 target protein Efg1 is likely to occur in the nucleus since Efg1 has been detected exclusively in the nucleus [18]. In *Saccharomyces cerevisiae*, activated PKA and the mitogen-activated protein kinase (MAPK) Hog1 were found to associate with promoters and coding regions of genes regulated by these kinases [19–21]. Action of the Hog1 MAPK on the Sko1 trancriptional repressor required the activity of kinase Sch9, which is structurally related to PKA, on the promoters of target genes [22]. The latter findings suggested that also in *C. albicans*, Tpk isoforms and possibly other kinases reside on genes that represent downstream targets of PKA signalling during hyphal morphogenesis. In this study, we strengthen this concept by demonstrating that PKA isoforms reside on specific genomic locations that change dramatically during morphogenesis from ORF to promoter regions. During the yeast-hyphal transition, genomic Tpk2 binding sites identify genes with known functions in dimorphism and suggest the identity of new genes involved in this cellular differentiation process.

## Results and discussion

### *C. albicans* strains producing HA-tagged PKA kinases

**Figure 1:**
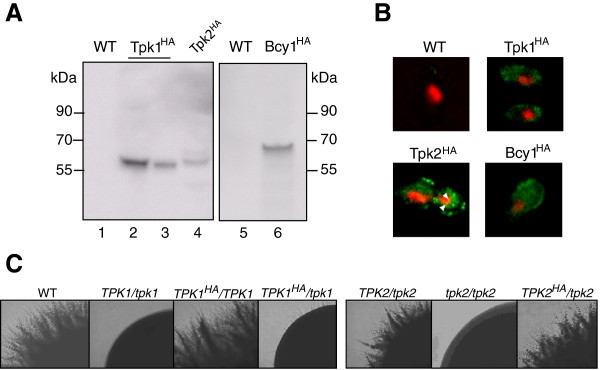
**Epitope-tagged PKA in** ***C. albicans.*** **A**. Immunoblotting to detect HA-tagged PKA subunits. Strains were grown in liquid YPD medium to OD_600_ = 0.6-0.8 and proteins in cell extracts (50 μg) were separated by SDS-PAGE (10% acrylamide), blotted onto PVDF membranes and probed using anti-HA antibody. Strains tested were CAF2-1 (WT: lanes 1, 5), AF1003 (*TPK1* ^*HA*^: lane 2), AF1004 (*TPK1* ^*HA*^: lane 3), AF1005 (*TPK2* ^*HA*^: lane 4) and AF1007 (*BCY1* ^*HA*^: lane 6)*.* **B**. Fluorescence microscopy of transformant cells treated with rat anti-HA antibody, which was detected with FITC-labelled anti-rat antibody (green signal) and DAPI to detect nuclei (red signal); yellow overlay signals are indicated by white triangles. **C**. Filament formation of strains producing HA-tagged Tpk proteins. Strains CAF2-1 (wt), II (*TPK1/tpk1*), TPK7 (*TPK2/tpk2*), TPO7.4 (*tpk2/tpk2*), AF1003 (*TPK1* ^*HA*^ */TPK1*), AF1004 (*TPK1* ^*HA*^ */tpk1*) and AF1005 (*TPK2* ^*HA*^ */tpk2*) were grown for 3 d/37°C on Spider agar and single colonies were photographed.

The single remaining allele encoding the catalytic subunits of PKA kinase was modified in heterozygous *C. albicans* mutants to add sequences specifying a C-terminal triple hemagglutinin (HA) epitope tag. Immunoblotting revealed the presence of HA-fusions to Tpk1 and Tpk2 proteins in cellular extracts of these strains during yeast growth (Figure 1A). Immunofluorescence microscopy revealed that the majority of fusion proteins resided in the cytoplasm of cells, while a minor fraction of Tpk2^HA^ was also detectable at the inside rim of the nucleus (Figure 1B, yellow dots). Similar Tpk cellular localization was observed in cells that were briefly (30 min) induced by 10% serum to form hyphae (data not shown).

To verify that HA fusion proteins were functional in the constructed strains we tested their filamentous growth, which is known to be regulated by the activity of Tpk1 and Tpk2 proteins [2, 3]. Inactivation of a single *TPK1* allele abolishes hyphal growth [3] but the *TPK1*^*HA*^*/TPK1* transformant formed hyphae as the wild-type strain (Figure 1C) indicating that the Tpk1^HA^ fusion protein is functional. Both *TPK2* alleles need to be inactivated to prevent hyphal growth [2]; therefore, it was verified that the filamentation phenotype of the *TPK2*^*HA*^/*tpk2* strain mimicked the *TPK2*/*tpk2* strain but not the *tpk2*/*tpk2* homozygous mutant (Figure 1C). This result shows that the Tpk2^HA^ fusion protein is functional. In summary, use of HA-tagged Tpk proteins revealed that in *C. albicans* as in fission yeast [15] the majority of PKA is located in the cytoplasm. The exclusive localization of a possibly non-functional Tpk1-GFP fusion within the nucleus [5] was not confirmed by the HA-tagged Tpk1 protein.

### Genomic localization of Tpk proteins

The above results suggested that a minor fraction of cellular PKA catalytic subunits resides in the nucleus of *C. albicans* cells. Furthermore, the presence of PKA isoforms and other kinases at target genes had been demonstrated previously in *S. cerevisiae*[19–21]. To verify, if nuclear PKA isoforms bind specific genomic targets in *C. albicans* we performed ChIP chip experiments with strains containing HA-tagged PKA isoforms; strains producing authentic non-tagged Tpk proteins were used as reference strains. Tpk1 and Tpk2 localization was examined during yeast growth or alternatively, following a brief period (30 min) of hyphal induction by 10% serum. During this time period, early regulatory processes take place that reprogram cells to allow hyphal growth. This became evident in wild-type cells by the formation of germ tubes after 30–60 min of induction.

**Figure 2:**
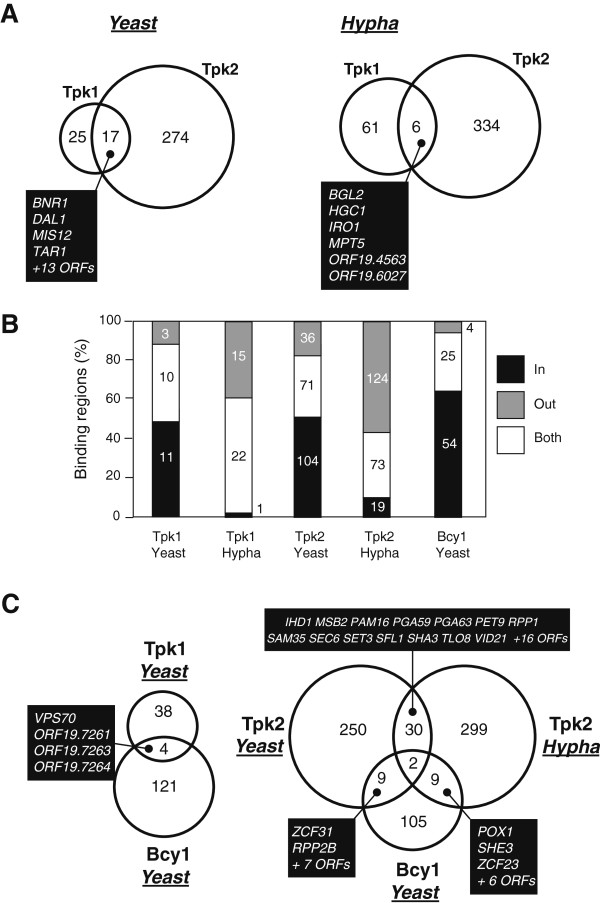
**Genomic localization of PKA subunits. A**. Genomic binding of PKA subunits. Venn diagrams number genes containing Tpk1 or Tpk2 binding sites in their promoter or ORF sequences; cells were grown in the yeast form or induced 30 min for hypha formation using 10% horse serum. Names of genes in the Tpk1/Tpk2 intersection are listed. **B**. Genomic PKA subunit localization. For each subunit, numbers of binding sites inside an ORF (black box), outside an ORF (promoter) (grey box) or at both sites (white box) are listed. **C**. Genomic binding of Tpk1, Tpk2 and Bcy1 subunits. The Venn diagram numbers indicate genes associated with the Tpk1 or Tpk2 catalytic subunit under yeast growth or hyphal induction, as well as the localization of the Bcy1 regulatory subunit during yeast growth. Gene names in the intersections are indicated.

A significant number of Tpk genomic binding sites was identified in both yeast and hyphal cells in duplicate ChIP chip experiments (see Additional file 1: Tables S1-S4). About tenfold more Tpk2 than Tpk1 binding sites were detected during yeast growth but Tpk1 binding increased during hyphal induction. A particular importance of the Tpk2 isoform for *C. albicans* morphogenesis under liquid growth conditions was reported previously [3]. The most significant peaks, which were overlapping in both replicates, were defined as binding sites. These sites were associated with ORF and promoter sequences of target genes (promoter binding sites within two divergently transcribed genes were assigned to both genes). Analyses indicated that very rarely, both Tpk1 and Tpk2 bind to same gene (Figure 2A). An exception is *HGC1* encoding an important regulator of hypha formation [23], which bound both Tpk1 and Tpk2 during hyphal induction. This result indicates that both catalytic PKA isoforms have mostly different genomic targets.

Importantly, during yeast growth both Tpk1 and Tpk2 were bound mostly within ORFs of target genes, while hyphal induction reduced ORF binding and favoured promoter binding or joint promoter-ORF binding (Figure 2B). Under yeast and hyphal growth conditions, all Tpk1 target genes and the majority of Tpk2 target genes were different (Figure 2C). Thus, ORF-to-promoter switching rarely occurs on the same gene during yeast-hyphal morphogenesis.

Growth of *C. albicans* in rich medium does not trigger hyphal formation, because PKA activity is repressed by the regulatory PKA subunit Bcy1 [5]. To test if during yeast growth both Tpk isoforms and Bcy1 bind to ORFs a ChIP chip experiment was performed on a *BCY1*^*HA*^*/BCY1* strain (AF1007). Immunoblotting showed the production of HA-tagged Bcy1 in transformants (Figure 1A) and immunofluorescence demonstrated that this protein resides mainly in the cytoplasm (Figure 1B). The Bcy1^HA^ fusion produced by transformants is functional because the *BCY1*^*HA*^/*BCY1* strain was insensitive to heat shock (2 h at 50°C) (data not shown), unlike a *BCY1*/*bcy1* strain [4]. Data analyses revealed a moderate number of genomic Bcy1 binding sites during yeast growth (see Additional file 1: Table S5). However, binding sites did not coincide to a great extent with Tpk1 or Tpk2 binding sites, either during yeast growth or during hyphal induction (Figure 2C). These results indicate that in general, Tpk1 or Tpk2 isoforms do not bind to target ORFs in the form of Tpk-Bcy1 holoenzyme complexes. Conceptually, ORF-bound Tpk proteins could either be active because of the absence of Bcy1 or their activities may be regulated by yet unknown mechanisms.

### Gene ontology analysis of PKA binding sites

**Figure 3:**
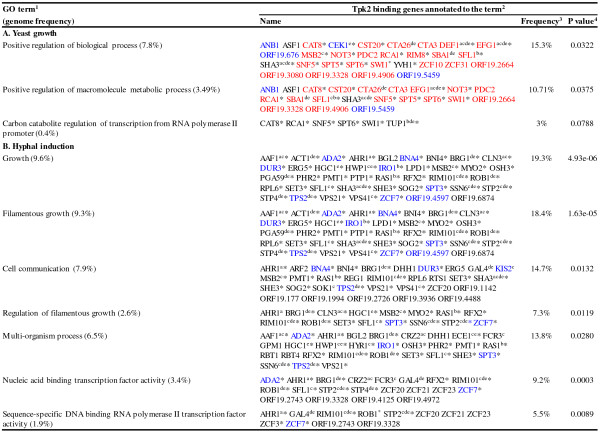
**GO terms of genes associated with Tpk2 binding sites.** **A.** Tpk2 binding sites during yeast growth. **B**. Tpk2 binding sites during hyphal induction. GO terms for Tpk2 binding targets were identified in ChIP chip data using the CGD GO Term Finder tool (http://www.candidagenome.org/cgi-bin/GO/goTermFinder). ^1^Genomic frequencies of genes corresponding to specific GO terms are expressed as percentages (gene number relative to 6,525 genes in the *C. albicans* genome [24]. Analysis conducted in April 2013. ^2^Gene name or ORF19 nomenclature according to CGD [24]. Some genes were attributed to more than one GO term. Red lettering: Tpk2 binding in ORF; blue lettering: Tpk2 binding in promoter and ORF; black lettering: Tpk2 binding in promoter ^a, b, c^Efg1 binds to promoter regions in ^a^yeast growth, ^b^hyphal induction or ^c^biofilm induction [13, 25, 26]. ^d, e^Set3 binding in ^d^yeast or ^e^hyphae inducing conditions [27]. ^3^Percentages were calculated based on the number of genes in each GO category divided by the total number (196 genes for Tpk2 in yeast form and 218 genes for Tpk2 in hyphal inducing conditions). ^4^ *P* values for overrepresented categories were calculated using a hyper geometric distribution with multiple hypothesis correction according to the GO Term Finder tool website (http://www.candidagenome.org/help/goTermFinder.shtml). The *P* value cutoff used was 0.1. *Genes upregulated during hyphal induction [24, 28].

Gene ontology (GO) analyses [24] of genes close to or harbouring PKA binding sites revealed significant GO terms for Tpk2 kinase but not for the Tpk1 isoform in any growth condition. During yeast growth, Tpk2 bound preferentially to ORFs (or both ORF and promoters) of genes encoding components of the general transcription machinery (e. g. Asf1, Def1, Swi1, Spt6), in addition to ORFs encoding transcription factors involved in carbon source utilization (e. g. Rca1, Snf5, Tup1) or filamentous growth (e. g. Efg1, Tup1, Sfl1) (Figure 3A). Furthermore, ORFs of genes for components of signalling pathways leading to hyphal formation (e. g. Cek1, Msb2) were bound by Tpk2 during yeast growth (Figure 3A). During hyphal induction, Tpk2 bound mostly to promoter regions of genes involved in filamentous growth (40 genes including *HGC1*, *RAS1*) (Figure 3B). Tpk2 target sites included promoters of genes encoding 20 transcription factors directly binding DNA (mostly containing zinc finger motifs). In summary, Tpk2 genomic binding shows a distinctive pattern of binding to morphogenesis-related genes, specifically the binding to ORFs during yeast growth and to promoters during hyphal induction. Tpk2 target genes are often bound also by Efg1 [13, 25, 26] in promoter regions or by the Set3C histone deacetylase complex [27] within their ORFs (superscripts d, e, in Figure 3). Recently, direct binding of heat shock factor-type transcriptional regulators Sfl1 and Sfl2 to Efg1 has been demonstrated [12], a finding also associating the genomic localization of these regulators with Tpk2 binding sites. In summary, Tpk2 binding identifies genes with known functions in filamentous growth and predicts such functions for yet uncharacterized *C. albicans* genes (designated *ORF19.* in Figure 3).

### ORF binding during yeast growth

ORF binding by PKA isoforms occurred mainly during yeast growth and included two relevant *C. albicans* genes, *EFG1* and *MSB2. EFG1* is required for the initial phase of yeast-hyphal transition but it is downregulated rapidly by negative autoregulation to allow undisturbed morphogenesis [29]; downregulation requires the Tpk2 but not the Tpk1 PKA isoform [13]. *MSB2* encodes a membrane sensor for environmental cues leading to hypha formation via the Cek1 MAPK and its shed domain provides resistance to antimicrobial peptides [30, 31].

**Figure 4:**
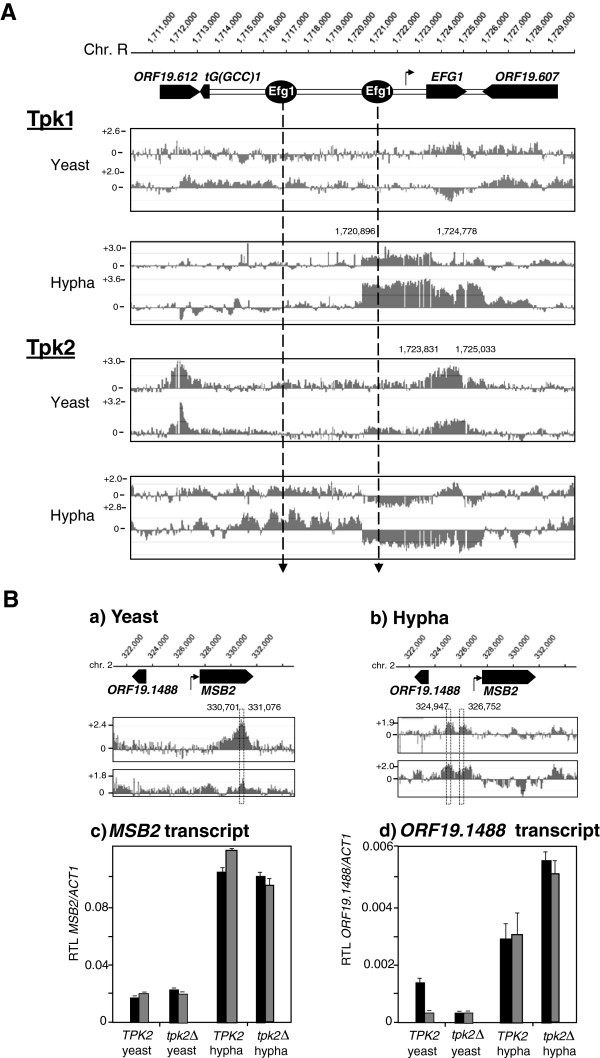
**Tpk binding to** ***EFG1*** **and** ***MSB2*** **loci. A**. Binding to *EFG1.* Coordinates of *EFG1* and neighbouring genes on chromosome R are shown on top. Round circles (dotted lines) indicate the position of Efg1 binding during growth in the yeast form [13]; the kinked arrow indicates the transcript start site. The genomic localization of Tpk1 and Tpk2 was determined by ChIP chip assays on strains containing HA-tagged Tpk proteins relative to an unmarked control strain. For Tpk1 localization, the strain pair II (*TPK1/tpk1*)/AF1004 (*TPK1-3× HA/tpk1*) and for Tpk2 localization, the strain pair TPK7 (*TPK2/tpk2*)/AF1005 (*TPK2-3× HA/tpk2*) was used in ChIP chip. Graphs represent duplicates of Tpk^HA^ occupancy at the *EFG1* locus, ordinates represent scaled log_2_ ratios. **B**. Binding to *MSB2.* Tpk2 enrichment in **(a)** the *MSB2* ORF of yeast cells or **(b)** in the *MSB2/ORF19.1488* upstream region of cells following hyphal induction; the most significant binding sequences are indicated by the dotted box. Transcript levels of *MSB2* **(c)** or *ORF19.1488* **(d)** in yeast or in induced cells were determined by qPCR in wild-type CAF2-1 (*TPK2*) or in *tpk2*Δ mutant cells (strain TPO7.4) growing in the yeast form or induced 30 min for hypha formation. Transcript levels relative to the *ACT1* transcript (RTL) are shown for two biological replicates (black and grey bars) indicating means and standard deviations for three technical replicates.

During yeast growth, Tpk2 but not Tpk1 was bound to the *EFG1* ORF. Binding was also observed to the upstream gene (*ORF19.612*) but not to the downstream gene (*ORF19.607*) of *EFG1* (Figure 4A). Similarly, Tpk2 but not Tpk1 bound the *MSB2* ORF in a distinct peak (Figure 4B, a). Comparisons of *MSB2* transcript levels in a wild-type strain and a homozygous *tpk2* mutant, grown in the yeast form, revealed no significant differences (Figure 4B, c). Similarly, *EFG1* promoter activity did not differ significantly between a wild-type and a *tpk2* mutant strain [13]. We conclude that Tpk2 residency at both target loci has no major influence on transcription/transcript levels, although subtle regulatory influences on gene expression cannot be excluded. A function of Tpk2 binding at ORFs is suggested by a distinct codon preference in the Tpk2 binding region (see below).

### Promoter binding during hyphal induction

During hyphal induction PKA isoforms bound preferentially to promoter regions. At the *EFG1* promoter extensive binding of Tpk1 but not of Tpk2 was detected (Figure 4A). The broad Tpk1 binding area ranges from the *EFG1* transcriptional start site through 1169 bp untranslated upstream sequences and ends close to the 3′end of the *EFG1* ORF. The Tpk1 binding area matches one of the major binding sites for Efg1 in the yeast form indicating that shortly after hyphal induction, Tpk1 binding occurs concomitant with the release of Efg1 [13]. *EFG1* promoter downregulation had been also observed in a *tpk1* mutant [13] suggesting that Tpk1 has no major role in negative autoregulation of *EFG1*.

**Figure 5:**
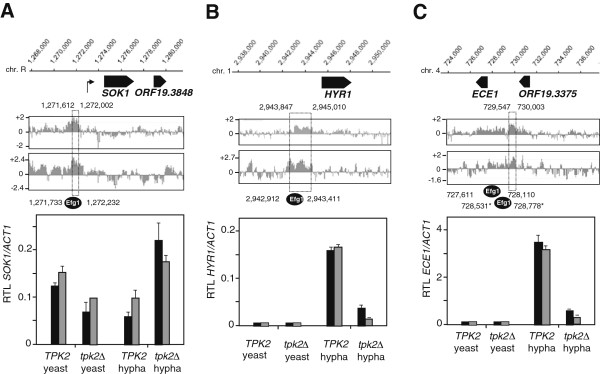
**Tpk2 promoter binding during hyphal induction.** Tpk2 localization was determined in cells induced for hypha formation at *SOK1* **(A)**, *HYR1* **(B)** and *ECE1* **(C)** genomic loci. ChIP chip occupancy values of Tpk2^HA^ were determined in duplicates as in Figure 4; peak binding areas are marked by a dotted box. All three genes contain Efg1 binding sites within their promoters (circles) at the indicated coordinates; positions marked were reported by Nobile *et al.*[25] and by Lu *et al.*[26] (asterisks). The kinked arrow indicates the *SOK1* transcript start site [24]. Transcript levels for each gene during yeast growth or hyphal induction were determined by qPCR as in Figure 4B.

During hyphal morphogenesis, binding of Tpk2 within promoter regions of several genes was correlated with transcript levels of these genes (Figure 5A-C). The transcript of the *SOK1* gene (encoding a putative stress-regulated kinase [32]) was downregulated during hyphal induction in wild-type strains, which did not occur but was even upregulated in a *tpk2* mutant (Figure 5A). The Tpk2 binding peak (coordinates 1,271,612-1,272,002) lies directly upstream of the transcriptional start site of *SOK1* at position 1,272,810 (Figure 5A, kinked arrow) [33]. During hyphal induction, Tpk2 binding in the intergenic region between *ORF19.1488* and *MSB2* genes did not affect the *MSB2* transcript but correlated with an increased level of the *ORF19.1488* transcript in the *tpk2* mutant (Figure 4B). Furthermore, transcript upregulation of *HYR1* and *ECE1* genes, which are expressed specifically in the hyphal growth form [34, 35], was completely abolished in the *tpk2*Δ strain (Figure 5B, C).

Promoter regions of *SOK1*, *HYR1* and *ECE1* genes are known to bind the Efg1 regulator relatively late during hyphal induction [26] or during biofilm formation [25], while no Efg1 binding was detected shortly (30 min) after hyphal induction [13]. Interestingly, Efg1 binding sequences are not identical but overlap partially with the sequences bound by Tpk2 (Figure 5A-C). Taken together, these results suggest that Tpk2 binding to promoters has the potential to regulate transcription of *C. albicans* genes both negatively and positively, possibly involving subsequent binding of Efg1 as a PKA phosphorylation target [9].

### Sequence motifs in Tpk2 binding regions

**Figure 6:**
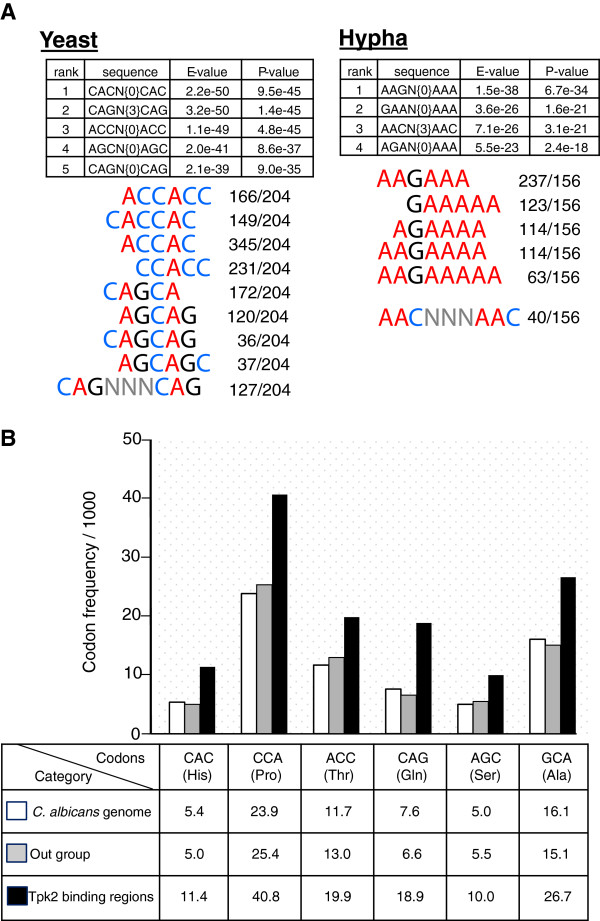
**Tpk2 binding specificity. A**. Consensus sequences for Tpk2 binding sites in yeast cells (left) or in cells induced for hypha formation (right). The program RSAT dyad-analysis [36] was used to predict consensus sequences. Predicted dyads (pairs of trinucleotides) common to Tpk2 binding sites were ranked and qualified by their *P-*/*E-*values; the frequencies of the deduced consensus sequences and of closely related sequences relative to all binding sites are listed below each table. **B**. Occurrence of trinucleotides/codons in ORF regions bound by Tpk2 during yeast growth. Codons corresponding to the Tpk2 binding consensus sequence are listed, along with codon frequencies in all *C. albicans* ORFs, in a randomly picked group of 150 genes not binding Tpk2 (out group) and in the 204 Tpk2 binding regions occurring mostly within ORFs.

Sequences representing the binding peaks of PKA were analysed for consensus sequences using the RSAT programs dyad-analysis and peak-motifs [36, 37]; both algorithms generated identical results. While no significant consensus motifs were detected for Tpk1, Tpk2 showed clear sequence preferences. If cells were grown in the yeast form, Tpk2 binding occurred most frequently within ORFs (Figure 2B) at ACCAC, CCACC or CAGC motifs (Figure 6A). During hyphal induction, however, when Tpk2 binds predominantly within promoter regions (Figure 2B), a completely different set of binding preferences was found (Figure 6A). The identified A_5_GA_5_ and A_2_GA_5_ motifs match the A_2_GA_5_ motif for binding of the Azf1 transcriptional regulator in *S. cerevisiae*, which is required for glucose-induced gene transcription [38]. Consistent with this activity, effective hypha formation in *C. albicans* is known to require low levels of glucose [39]. The identified AAC, AAG and ACC repeats, which were identified in most Tpk2 binding sites, had been also detected previously for the Efg1 transcriptional regulator during hyphal induction [13]. The *HYR1* and *ECE1* genes, which are induced by hypha formation [34, 35], contain these consensus sequence motifs, possibly to permit binding of kinase and its downstream target Efg1 to jointly trigger morphogenesis-dependent gene expression.

Binding of Tpk2 to many ORFs raised the question if such bound ORF sequences were as free as unbound regions to evolve sequence variants, e. g. with regard to the usage of synonymous codons. Therefore, we compared overall *C. albicans* codon usage with codon usage in ORF sequences bound by Tpk2. Specifically, we investigated if codons corresponding to the deduced Tpk2 binding consensus sequences would be preferred in the Tpk2 binding region. It was indeed found that usage of all six codons matching the Tpk2 consensus sequence during yeast growth was increased as compared to the average codon usage in *C. albicans* or to random set of 150 ORFs (“out group”) that are not bound by Tpk2 (Figure 6B). In the case of histidine even a complete reversal of codon usage from the preferred CAT codon (15.62/1000 to 8.63/1000) to CAC (5.39/1000 to 11.4/1000) was observed in the Tpk2 binding region. This result suggests that ORF binding Tpk2 has a vital, yet unknown function, because it exerts selective pressure to restrict codon usage within ORFs. Codon usage has hitherto been related mainly to abundance of aminacyl-tRNAs [40].

### PKA localization at the *EFG1* locus

**Figure 7:**
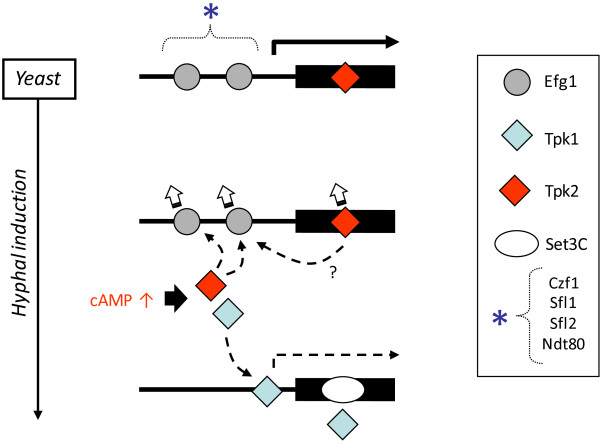
**Residency of PKA isoforms at the** ***EFG1*** **locus during hyphal induction.** During yeast growth, Efg1 [13] and Tpk2 proteins reside on promoter and ORF, respectively, and *EFG1* is transcribed at high levels (top). During initiation of hyphal formation, increased cAMP levels activate Tpk isoforms by removing Bcy1 repression and Tpk2 phosphorylates Efg1 [9]. This is followed by rapid removal of both Efg1 [13] and Tpk2 (indicated by open arrows). Subsequently the Tpk1 isoform enters the *EFG1* promoter at one of the previous Efg1 binding sequences and the Set3C histone deacetylase [27] occupies the ORF. Other proteins including Czf1, Sfl1, Sfl2 and Ndt80 are known to bind the *EFG1* promoter [12, 25] and may contribute to Tpk-dependent regulation of *EFG1* expression.

The *EFG1* gene is a paradigm of both PKA and Efg1 binding to a genomic locus (scheme of main events in Figure 7). Early during hyphal induction the *EFG1* transcript level is lowered rapidly, presumably because the continued presence of Efg1 disturbs hyphal morphogenesis [29]. *EFG1* downregulation requires the Efg1 protein and the Tpk2 PKA isoform [13] within a short time window to initiate hyphal formation, because both proteins leave the *EFG1* locus rapidly thereafter. Possibly, genomic binding of both proteins prepares yeast cells to undergo hyphal morphogenesis rapidly in inducing environments. Binding of the Tpk1 isoform at promoter and coding region of *EFG1*, as well as binding of the Set3C histone deacetylase complex to the ORF [27] may also help to establish the repressed state of *EFG1* during hyphal formation. *EFG1* expression is probably regulated by additional proteins binding the *EFG1* promoter directly or indirectly by adapter proteins including Efg1. Candidate Efg1-binding proteins localized to the *EFG1* promoter include Czf1, Flo8, Sfl1, Sfl2 and Ndt80 proteins [11, 12, 25]. Tpk2 binding to the *SET3* and *SFL1* promoters, which we observed (Figure 3), may support this activity. Co-operation of Tpk2 and Efg1 may also occur at other genes regulated by hyphal induction including *HYR1*, *ECE1* and *SOK1* genes, as described above. Thus, the proposed Tpk2 phosphorylation of Efg1 [9, 13] and other components of the transcriptional initiation machinery could occur directly at the promoters of genes regulating morphogenesis.

## Conclusions

PKA isoforms Tpk1 and Tpk2 are crucial for the virulence of the human fungal pathogen *C. albicans* by regulating dimorphic growth*.* Tpk proteins mediate environmental cues and trigger hyphal morphogenesis by altering the transcriptional program. We show that Tpk2 and to a lesser extent Tpk1 bind to specific genomic sequences within ORFs and promoters of target genes. Growth in the yeast form triggers Tpk binding to CA-rich sequences within ORFs and appears to bias codon usage within the binding region. During hyphal induction Tpk2 associates with promoter regions of genes regulating or regulated by hyphal morphogenesis, often proximal to binding sites for the Efg1 transcription factor. These results suggest that genomic PKA proteins facilitate and/or prolong hyphal morphogenesis by acting on nearby transcription factors at genes regulating morphogenesis. Molecular mechanisms of PKA nuclear import, genomic recruitment and function remain to be established. In conclusion, we have demonstrated for the first time in a fungal pathogen that PKA isoforms, which are responsible for a relatively simple developmental program in a single cell, mark downstream target genes. Such binding analyses have predictive value because they link yet uncharacterized genes to signalling by PKA isoforms. This concept may hold true for other cellular differentiation processes involving other types of kinases and other species.

## Methods

### Strains and growth conditions

*C. albicans* strains are listed in Table 1 Strains were grown in liquid or on solid YPD, YPS and supplemented SD minimal medium, as described [13]. To induce hyphae the strains were grown at 37°C in YP medium containing 10% horse serum. *C. albicans* strains producing C-terminally HA-tagged PKA isoforms were constructed by transformation of heterozygous strains retaining a single allele of the respective gene. *HA-URA3* tagging cassettes were PCR amplified using oligonucleotide pairs on template plasmid p3HA-URA3, which generated PCR products ending in homologous sequences to target the respective genes [41]. Oligonucleotides Tpk1-HA (for)/(rev) and Tpk2-HA (for)/(rev) were used to generate tagged strains AF1003 (*TPK1*^*HA*^/*TPK1*) from CAI4, AF1004 (*TPK1*^*HA*^/*tpk1*) from FII4a and AF1005 (*TPK2*^*HA*^/*TPK2*) from AF1001. Similarly, oligonucleotides Bcy1-HA (for)/(rev) were used to tag one of the *BCY1* alleles of strain CAI4 to generate strain AF1007 (*BCY1*^*HA*^/*BCY1*). Correct chromosomal integration of tagging cassettes was verified by colony PCR using primers TPK1ver, TPK2ver, BCY1ver in combination with primer 3′Test HA-tag. Oligonucleotides are listed in Additional file 2: Table S6.

**Table 1:** ***C. albicans*** **strains**

Strains		Reference
CAF2-1	*URA3/ura3*::*imm434*	[42]
CAI4	*ura3*::*imm434/ura3*::*imm434*	[42]
II	As CAI4 but *TPK1/tpk1::hisG-URA3-hisG*	[3]
FII4a	As CAI4 but *TPK1/tpk1::hisG*	[3]
AF1003	As CAI4 but *TPK1*::(*3xHA-URA3*)*/TPK1*	This work
AF1004	As FII4a but *TPK1*::(*3xHA-URA3*)*/tpk1::hisG*	This work
TPK7	As CAI4 but *TPK2/tpk2::hisG-URA3-hisG*	[2]
TPO7 (AF1001)	As TPK7 but *TPK2/tpk2::hisG*	[2]
TPO7.4	As CAI4 but *tpk2::hisG/tpk2::hisG-URA3-hisG*	[2]
AF1005	As AF1001 but *TPK2*::(*3xHA-URA3*)*/tpk2*::*hisG*	This work
AF1007	As CAI4 but *BCY1*::(*3xHA-URA3*)*/BCY1*	This work

### Immunodetection

Proteins containing a hemagglutinin (HA) antigenic tag were detected in cell extracts by immunoblotting using monoclonal rat anti-HA antibody (Roche; 1:1000), which was visualized on blots using peroxidase-coupled goat antibody (Pierce; 1:10000). Cells used for immunofluorescence microscopy were fixed by 4% formaldehyde and 1 ml of cell suspension were treated by zymolyase T100 (100 μg), glucuronidase (30 μl) and 10 mM DTT for 30 min at 30°C. Cells were pelleted and treated with 0.1% Triton X-100 for 5 min at room temperature. Cells (20 μl) were fixed to polylysine-coated glass slides and washed with PBS, followed by blocking of unspecific binding sites using 2% milk powder in PBS. The blocking solution was removed and 40 μl of rat anti-HA antibody (Roche; 1:100) were allowed to react 90 min at room temperature or overnight at 4°C in a wet chamber. Cells were washed and fluorescein isothiocyanate (FITC)-coupled goat anti-rat antibody (Jackson Immunologic Research Lab Inc.; 1:100) in 0.2% milk powder was added and allowed to react for 90 min at room temperature. For nuclear staining 20 μl diamidino-2-phenylindole (DAPI; 1 μg/ml) was added for 15 min at room temperature. Slides were washed by PBS and a drop of anti-fade (Pro-Long Anti-Fade, Sigma) was added before covering the specimen with a cover slip, which was sealed by nail polish. Microscopic inspection of FITC and DAPI fluorescence was done using a spinning disc confocal microscope (Cell Observer® SD; Yokogawa CSU-X1) and using the program Zen 2011 (Carl Zeiss) for evaluation of images.

### Chromatin immunoprecipitation on microchips (ChIP chip)

The ChIP chip procedure was carried out essentially as described [13] except that magnetic beads with bound antibodies were eluted twice with elution buffer for 20 min at 65°C and that RNA was removed by adding 2.5 μl of RNase A (10 mg/ml; Qiagen). *C. albicans* genomic tiling microarrays were probed pairwise by immunoprecipitated chromatin of a strain producing an HA-tagged protein and a corresponding control strain. The following pairs of strains were used: II (*TPK1/tpk1*)/AF1004 (*TPK1-3× HA/tpk1*), TPK7 (*TPK2/tpk2*)/AF1005 (*TPK2-3× HA/tpk2*), CAF2-1 (*BCY1/BCY1*)/AF1007 (*BCY1-3× HA/BCY1*). Two independent cultures were assayed for each combination of strains. Significant binding peaks were defined as probes containing four or more signals above background in a 500 bp sliding window; the degree of significance depended on the FDR value. Results were visualized using the program SignalMap (version 1.9). The most significant binding peaks (FDR ≤ 0.05), which coincided in both replicates, were analyzed by the program RSAT dyad-analysis to predict binding sequence from all peak genomic binding sites [36]. Codon usage of all *C. albicans* genes was derived from the Candida Genome Database [24] and codon usage in sequences of ORFs bound by Tpk2 were calculated using the Codon Usage Calculator [43].

### Availability of supporting data

The data sets supporting the results of this article are available in the Candida Genome Database (CGD) repository: http://www.candidagenome.org/download/systematic_results/Schaekel_2013/.

## Electronic supplementary material

Additional file 1: Table S1: Lists Tpk1 binding sites during yeast growth. **Table S2.** Lists Tpk1 binding sites during hyphal induction. **Table S3.** Lists Tpk2 binding sites during yeast growth. **Table S4.** Lists Tpk2 binding sites during hyphal induction. **Table S5.** Lists Bcy1 binding sites during yeast growth. (XLS 212 KB)

Additional file 2: Table S6: List of oligonucleotides. (DOC 32 KB)
