# Supplementary material for: Morphogenesis-regulated localization of protein kinase A to genomic sites in Candida albicans
Source: BMC Genomics. 2013 Dec 1;14(1):842. doi: 10.1186/1471-2164-14-842 (PMC4046665; doi:10.1186/1471-2164-14-842)
Supplement: Supplementary file 2 — Additional file 2: Table S6: List of oligonucleotides. (DOC 32 KB) [file 12864_2013_5556_MOESM2_ESM.doc]

**Table S6. List of oligonucleotides**

| name | sequence |
| --- | --- |
| Tpk1-HA (for) | 5´- ATTTGATCGATACCCAGAAGATAAAGATTTGGATTATGGTATAAGTGGAGTTGAAGACCCATATCGTGATCAATTCCAGGACTTTGGTGGTGGTCGGATCCCCGGGTTAATTAA-3´ |
| Tpk1-HA (rev) | 5´-CAATTTAACAAGGAAGCCAATTCATATAGTATCATTGGTTTAAAAACAAGACACTGCTTATATTTTAGCTGTCTAGAAGGACCACCTTTGATTG-3´ |
| Tpk2-HA (for) | 5´-GATTCGTCATTGTTTGACCATTATCCAGAAGAACAATTAGACTACGGAAGCCAAGGAGAAGATCCTTATGCTCTGTATTTCCTTGACTTTGGTGGTGGTCGGATCCCCGGGTTAATTAA-3´ |
| Tpk2-HA (rev) | 5´-CCGAAAAACAAAGCAGACAAGGAAGAGTCAACATGTTAAGTCATTCGTATAATAACAAAACGAAAATATTAATGTGCTCTAGAAGGACCACCTTTGATTG-3 |
| Bcy1-HA (for) | 5´-TAAATCCGGGTTCCAAAGATTATTGGGTCCTGTTGTGGAGGTATTGAAAGAACAAGACCCTACAAAGAGTCAAGACCCAACTGCTGGTCATGGTGGTGGTCGGATCCCCGGGTTAATTAA-3´ |
| Bcy1-HA (rev) | 5´-CTCGTTCATGCTGCCATCTCGTATATACCTGACAACAAGAAATGTAGTTGAACTTTTGCACCAACCATCCATTATCAAAATCGCAACGAAAACACCATCTAGAAGGACCACCTTTGATTG-3´ |
| TPK1ver | 5´-AGTTGATTGGTGGTCATTTG-3´ |
| TPK2ver | 5´-ATCTATCGTGATTTGAAACC-3´ |
| BCY1ver | 5´-ATTAGACCGTTTGACATTCC-3´ |
| 3‘ Test HA-tag | 5´-CATCGTATGGGTAAAAGATG-3´ |
